# Supplementary material for: Epidemiology of Schistosoma mansoni infection and associated risk factors among school children attending primary schools nearby rivers in Jimma town, an urban setting, Southwest Ethiopia
Source: PLoS One. 2020 Feb 27;15(2):e0228007. doi: 10.1371/journal.pone.0228007 (PMC7046261; doi:10.1371/journal.pone.0228007)
Supplement: S1 Questionnaire — (DOCX) [file pone.0228007.s001.docx]

JIMMA UNIVERSITY

INSTITUTE OF HEALTH

FACULTY OF HEALTH SCIENCES

SCHOOL OF MEDICAL LABORATORY SCIENCES

**І .Questionnaires used to study epidemiology of *S. mansoni* infection and associated risk factors among school children in five selected primary school nearby rivers in Jimma town, Southwest Ethiopia (English Version).**

**Introduction**

My name is Azalech Tefera from Jimma University; I am here to study about *Schtosoma mansoni* infection and associated risk factors among school children in selected primary schools.

The main objective of this study is to determine epidemiology of *Schistosoma mansoni* infection and its associated risk factors among school children in selected primary schools nearby rivers in Jimma Town, Southwest, Ethiopia. The questionnaire is prepared to evaluate associated risk factors for *Schistosoma mansoni* infection.

I am asking you to participate in the study for *Schistosoma mansoni* infection investigation. The investigation will involve collection of feces for parasitological examination and interview through pre-structured questionnaire for socio-demographic characteristics and associated risk factors for *S. mansoni* infection.

If the result of investigation is positive for *Schistosoma mansoni* infection, you will be treated with appropriate drug and it is your right not to involve in study if you are not interested and withdraw at any time from the study. Finally, if you have understood the explanation very well I am asking you kindly to participate in this study, and put your signature below.

It is with my full understanding I agreed to give the informed consent voluntarily to researcher to participate in the study.

Signature (participant) __________ Date___________

Signature (Investigator) _________ Date __________

Thank you for your participation

Survey questionnaire used for assessment of associated risk factors for *Schistosoma mansoni* infection among school children in selected primary schools nearby rivers in Jimma town southwest Ethiopia.

Questionnaire part I; - Socio-demographic characteristics of the schoolchildren.

| S/no. | Quesstions | Response | Remark |
| --- | --- | --- | --- |
| 101 | Age |  |  |
| 102 | Gender | 1. Male  2. Female |  |
| 103 | School | 1. Seto Yiddo 4. Jimma  2. Tesfa Tewahido 5. Kitto  3. Hamile 19 |  |

Questionnaire part II:-This questionnaire is proposed to assess clinical history and physical examination of schoolchildren related to *Schistosoma mansoni* infection**.**

| S/no. | Questions | Response | Remark |
| --- | --- | --- | --- |
| 201 | Abdominal pain / discomfort | 1. Present 2. Absent |  |
| 202 | Palpable hepatomegaly | 1. Present 2. Absent |  |
| 203 | Palpable splenomegaly | 1. Present 2. Absent |  |
| 204 | Diarrhea | 1. Present 2. Absent |  |
| 205 | Fever | 1. Present  2. Absent |  |
| 206 | Weakness | 1. Present  2. Absent |  |

Questionniare Part III: - This questionnaire is intended to assess risk factors related to *Schistosoma mansoni* infection.

|  | Questions | Response | Remark |
| --- | --- | --- | --- |
| 301 | Source of water for drinking | 1. Private tap  2. Public tap  3. River  4. Others______ |  |
| 302 | Source of water for cooking | 1. Private tap  2. Public tap  3. River  4. Others______ |  |
| 303 | If you bring water for cooking from river, from where do you fetch it? | 1. Awetu  2. Kitto  3. Setto  4. Keba |  |
| 304 | Do you defecate nearby river? | 1. Yes 2. No |  |
| 305 | Do you have latrine? | 1. Yes 2. No |  |
| 306 | If you answered Q. 305 yes, do the whole families use it? | 1. Yes 2. No |  |
| 307 | If you answered Q. 305 no, where do you defecate and dispose the feces? | 1. Near the river  2. Away from the river  3. Using pits and dispose to the river |  |
| 308 | Do you swim in nearby river? | 1. Yes 2. No |  |
| 309 | If yes answered Q 308, where do you swim? | 1. Awetu  2. Kitto  3. Setto  4. Keba  5. Others__________ |  |
| 310 | How often you swim in the river? | 1. Frequently  2. Often  3. Sometimes |  |
| 311 | Do you have contact to the river while you are crossing it? | 1. Yes  2. No |  |
| 312 | If you answered yes to Q. 311, which river? | 1. Awetu  2. Kitto  3. Setto  4. Keba  5. others |  |
| 313 | Do you wash cloths in the river? | 1. Yes 2. No |  |
| 314 | If you answered yes to Q. 313, where? | 1. Awetu  2. Kitto  3. Setto  4. Keba  5. others |  |
| 315 | Where do you bath? | 1. River  2. home |  |
| 316 | If the answer to Q. 315 is 1 then which river? | 1. Awetu  2. Kitto  3. Setto  4. Keba  5. others |  |
| 317 | If the answer to Q. 315 is 2 from where do you obtain the water? | 1. River  2. Pipe  3. Spring protected  4. Well protected |  |

**Yuniiversitii Jimmaa**

**Instiituutii Fayyaa**

**Fakkaaltii Saayinsii Fayyaa**

**Mummee Saayinsii Laboraatoori Medikaalaa**

**Questionnaire (Afaan Oromo Version)**

**Seensaa**

Kayyoon guddan qorannichaa haalli tamsa’ina fi ciminaa dhukkuba Bilaaharziiyaa mar’imaanii ijoollee mannen baruumissa nanno lagatti dhi’ataanii jiran irrattii sakatta’uufi.

Akkasumas gaafannoon kun wantoota dhukkuba bilaaharziiyaa mar’imaanii namatti fidan irrat hubanno fi ilaalchaa uumatin qabu madaaluuf kan qopha’eedha. Kana keessatti hirmannan keessan baay’ee barbachisaadha.Yoo keessatti hirmaachudhaaf fedhii qabatan waali galttan, ta’ee boolii guddaa qoodaa laastikii qoopha’ee jiru kanatti isin irra argachuun barbada, kunis jiirachuu bilaharziiyaa mar’immanii keessaa sakkata’uf ta’a. Qoranno kana keessatti hirmachuun keessan balaa isinttii fiduu tokko ille hin qabuu.Yoo dhuukubin kun isin keessatti argaame qorchaa isaa yaaluf ta’uu kafaaltii tokko malee tolan yaalmtuu. Oddeefannon isin kennitan dhimma qorannoo kanaatiif qofa kan fayyadamnnu fi qaama birootti kan hin himamne wan ta’eef soda tokko illee hin qabatinaa. Itti dabalees qorannicha keessati hirmaachuu dhiisunii fi yeroo barbaaaddan addaan kutuun mirga keessani.Hirmaannaa keessaniif isin galateeffanna.

Qorannicha keessatti hirmaachuuf fedhii guutuu qabdduu?

1. Eyyee

2. Lakki

Guyya _______________

Maqaa gaafatichaa ________________

Maqa to’ataa ___________________

Maqaa mana Barumssa ____________________

Af-gaaffii1ffaa qoratadhan dursamuun haalota faalama Bilaaharziiyaa mar’imaaniif nama saaxilan qorachuuf barattotaa mannen barnoota sadarkaa 1^ffa^ magaala Jimmaa keessatti adeemsifamu.

Gaaffilee filanoo armaan gadiitif deebii hirmatoota itti mari.

Af-gaaffii kutaa tokkoffa:- Halaa hawaasa-dinagddee baratoota.

|  | Gaaffilee | Deebii | Yaada |
| --- | --- | --- | --- |
| 101 | Umurii |  |  |
| 102 | Saala | 1. Dhiira 2. Dhalaa |  |
| 103 | Mana barumssa | 1. Yiddo Saxoo 4. Jimmaa 2. Tasfatawado 5. Kittoo 3. Hamilee 19 |  |

Af-gaaffii kutaa 2^ffa^:- Gaaffileen kun haloota faalama Bilaaharziiyaa mari’maanii seena fi qama

ijoollee sakata’udhan walttii dhufeenyaa isaani qorachuu.

|  | Gaaffilee | Deebii | yaada |
| --- | --- | --- | --- |
| 201 | Garaa dhukubbi / gidiiraa | 1.Jira  2.Hinjiru |  |
| 202 | Dhiitoo tiruu | 1.Jira  2.Hinjiru |  |
| 203 | Dhiitoo rajoo/rajjiji | 1.Jira  2.Hinjiru |  |
| 204 | Garaa-yaasaa/kaasa | 1.Jira  2.Hijiru |  |
| 205 | Qaama gubaa | 1.Jira  2.Hinjiru |  |
| 206 | Humnna dhabuu | 1.Jira  2.Hijiru |  |

Af-gaaffi kutaa 3^ffa^: - gaffileen kun haloota falama Bilaaharziiyaa mar’imanii wajjin hidhata qaban qorachuuf qophaa’ani.

|  | Gaaffilee | Deebii | Yaada |
| --- | --- | --- | --- |
| 301 | Bishaan dhugaatii eessaa fayyadamtuu? | 1.Boombaa dhunfaa  2. Boomba uummata 3.Bishaan bokka  4.laga  5.Kan biroo______ |  |
| 302 | Bishaan nyaata qophessuf eessa arggattu? | 1.Boombaa dhunffaa  2. Boomba uummata  3.laga  4.Kan biroo______ |  |
| 303 | Bishaan nyata qopheessuuf yoo laga fiddan, eessa warabddu? | 1.Haweetuu  2.Kitto  3.Saxoo |  |
| 304 | Laga bukkeetti ni boolatu? | 1.Eyyee  2.Lakki |  |
| 305 | Mana fincaani qabddu? | 1. Eyyee 2. Lakki |  |
| 306 | Yoo deebiinkeessan gaffi 305”Eyyee”ta’e, matiin hunduumtu ni fayyadamu? | 1. Eyyee  2. Lakki |  |
| 307 | Yoo deebiinkeessan gaaffi 305 “Lakki” ta’e, eessatti fayyadamttu ? | 1.Nanno laga  2.Laga irra fagaate  3.Boolla fayyadamu fi lagatti gatuu. |  |
| 308 | Laga nannookeessan jiru nidakituu? | 1. Eyye  2. Lakki |  |
| 309 | Deebiinkeessan gaaffi 308“Eyyee”yoo ta’e, laga isa kam daktuu ? | 1.Haweetuu  2.Kitto  3.Saxoo  4.Kanbiroo_____________ |  |
| 310 | Laga yeeroo meeqa daaktuu? | 1. Yeeroo baa’ee  2.Yeeroo hunda  3.Darbedarbe |  |
| 311 | Yoommu laga qaxamurttan qaminkeessan bishaan hintuqa? | 1.Eyyee  2.Lakki |  |
| 312 | Deebiinkeessan gaaffi 311 “Eyye” yoo ta’e ,laga isa kam ? | 1.Haweetu  2.Kitto  3.Saxoo  4.Kabiro_____________ |  |
| 313 | Wayyaa lagatti micittuu? | 1. Eyyee 2. Lakki |  |
| 314 | Deebiinkeessan gaaffi 313 “Eyyee” yoo ta’e lagaa isa kami ? | 1.Haweetu  2.Kitto  3.Saxoo  4.Kabiroo_____________ |  |
| 315 | Essatti qaamaa keessan dhiqatu? | 1.Laga  2. mana  3.Lafa uummanni itti dhiqatu |  |
| 316 | Deebiinkeessan gaffi 315 1 yoo ta’e laga isa kam ? | 1.Haweetu  2.Kitto  3.Saxoo  4.Kabiroo barreessi_____________ |  |
| 317 | Deebiinkee gaffi 315 2 yoo ta’e bishaan essa arggattuu? | 1.Laga  2.Boombaa  3.Burqituu  4. Boolla |  |

ÏT ¿’>y`c=+

¾I¡U“ dÃ”e ›=”eƒƒ¿ƒ

¾I¡U“ Lx^„] dÃ”e ƒ/u?ƒ

**Annex III**: - Questionnaire (Amharic version)

SÓu=Á

¾²=I U`U` ª“ ›LT ¾wLG`²=Á uc} u¨”´ ›p^u=Á uT>Ñ–< ƒUI`ƒ u„‹ uI×“ƒ }T]­‹ SGŸM e`ß~”“ ¾T>ÁÅ`c¬ Ñ<Çƒ U” ÁIM ›”ÅJ’“ ›”ÉG<U ›ÒLß U¡”Á„‹” ÃÇedM ::u}ÚT] K²=I Ø“ƒ ÁÑKÓM ²”É ¾cÑ^ “S<“ ŸMÐ­ ¾T>¨cÉ ÃJ“M eK²=I ¾›`f­ MÏ u²=I ¾Ø“ƒ U`U` Sd}ñ uU`U` ¬Ö?ƒ LÃ“ uÏT Ÿ}T ¬eØ ¾wLG`²=Á ubq KSk’e ¾ÔL ›e}­Ó ›K¬:: uSÖ¾l ¨pƒ ¾T>cÖ<ƒ SMf‹ ˆ“ ›e}Á¾„‹ uS<K< uUeÖ=` ¾}Öul ÃJ“K<:: ÃG<” ›”³ ¾T>cÖ<ƒ SMf‹ G<K< ›¬’ƒ’ƒ ÁL†¬ ^J’< KU`U\ u×U ÖnT> “†¬:: u²=I ¾Ø“ƒ U`U` LKSŸðM“ uSGM uT”—¬U Ñ>²? KTqU Swƒ­ ¾}Öuk ’¬:: eK²=I ›eŸG<” ¾}vK¬” Ó”³u? ¬eØ ›eÑw}¬ SMŸU õnÅ˜’ƒ­” uò`T­ ›”Ç=ÑMÖ<M˜ ›ÖÃnKG<::

uØ“ƒ“ U`U` uSd}ð­ u×U ›“ScÓ“K”::

u²=I ¾Ø“ƒ“ U`S` KSd}õ õnÅ— •­ƒ ; 1.›­ 2. ›ÃÅKU

k” -------------

¾}ÖÁm¬ eU ---------------------------- ò`T -------------

¾}q××] eU --------------------------- ò`T ---------------

¾ƒUI`ƒ u~ eU ---------------------

¾}ÖÁm¬ SKÁ lØ`------------------



¾}Ö¾k¬” SMe Ÿ}²[²\ƒ ›T^à‹ Á¡wu<ƒ

SÖÃp ¡õM 1:- TIu^©“ Y’ Q´v© S[Í­‹

|  | ØÁo | SMe | ›e}Á¾ƒ |
| --- | --- | --- | --- |
| 101 | °ÉT@ |  |  |
| 102 | Óq | 1. ¨”É 2. c?ƒ |  |
| 103 | ƒ/u?ƒ | 1.ÃÊ cÙ  2.}eó }ªIÊ  3. NUK? 19 4. ÏT  5. ¡„ |  |

SÖÃp ¡õM2:- ÃI SÖÃp ¾wLG`²=Á uiq u›‹M LÃ ¾T>ÁS×¬” ‹Ó` ˆ“ UM¡„‹” ÃÇedM::

| 201 | ¾JÉ ISU / ‹Ó` | 1. ›K  2. ¾KU |  |
| --- | --- | --- | --- |
| 202 | ¾Ñ<uƒ ˆwÖƒ | 1. ›K  2. ¾KU |  |
| 203 | ¾×òÁ ˆwÖƒ | 1. ›K  2. ¾KU |  |
| 204 | }pTØ | 1. ›K  2. ¾KU |  |
| 205 | ƒŸ<dƒ | 1. ›K  2. ¾KU |  |
| 206 | É‹U | 1. ›K  2. ¾KU |  |
|  |  |  |  |

SÖÃp ¡õM 3:-ÃI SÖÃp KwLG`²=Á uiq ›ÒLß U¡”Á„‹” ÃÇedM::

|  | ØÁo | SMe | ›e}Á¾ƒ |
| --- | --- | --- | --- |
| 301 | KSÖØ ¬H Ÿ¾ƒ ÁÑ—K< ; | 1. ¾ÓM v”v  2. ¾Q´w v”v  3. ¨”´  4. K?L |  |
| 302 | UÓw KT²ÒËƒ ¬H Ÿ¾ƒ ÁÑ—K<; | 1. ¾ÓM v”v  2. ¾Q´w v”v  3. ¨”´  4. K?L |  |
| 303 | UÓw KT²ÒËƒ ¬H Ÿ¨”´ ÃkÇK<;ŸkÆ Ÿ¾ƒ—¬ ¨”´; | 1. ›«~  2. ¡„  3. cÙ  4. Ÿv  5. K?L |  |
| 304 | ¨”´ Ç` ÃìÇÇK<; | 1. ›­  2. ›ÃÅKU |  |
| 305 | i”ƒ u?ƒ ›K­ƒ; | 1. 1.›­ 2.›ÃÅKU |  |
| 306 | KØÁo lØ` 305 SMf­ ›­ ŸJ’ &G<K<U u?}cw ÃÖkTK<; | 1.›­ 2.›ÃÅKU |  |
| 307 | KØÁo lØ` 305 SMf­ ›ÃÅKU ŸJ’ ¾ƒ ÃÖkTK< ; | 1.¨”´ ›p^u=Á  2.Ÿ¨”´ ^p wKA  3.Ñ<ÉÕÉ SÖkU ›“ ¨”´ S×M |  |
| 308 | ¨”´ Ãª—K<; | 1.›­ 2. ›ÃÅKU |  |
| 309 | KØÁo lØ` 308 SMf­ ›­ ŸJ’& ¾ƒ Ãª—K<; | 1. ›«~  2. ¡„  3. cÙ  4. Ÿv  5. K?L |  |
| 310 | U” ÁIM Ñ>²? ¬H Ãª—K<; | 1. ²¨ƒ`  2. w²< Ñ>²?  3. ›Mö-›Mö |  |
| 311 | ¨”´ c=Ás`Ö< ¬H ’¡„ƒ Á¬nM; | 1.›­  2.›ÃÅKU |  |
| 312 | KØÁo lØ` 309 SMf­ ›­ ŸJ’& ¾ƒ—¬ ¨”´; | 1. ›«~  2. ¡„  3. cÙ  4. Ÿv  5. K?L |  |
| 313 | Mwf­” ¨”´ ÁØvK<; | 1.›­  2.ÃÅKU |  |
| 314 | KØÁo lØ` 311 SMf­ ›­ ŸJ’& ¾ƒ—¬ ¨”´; | 1. ›«~  2. ¡„  3. cÙ  4. Ÿv  5. K?L |  |
| 315 | ÑL­” ¾ƒ ÃqÖvK<; | 1.¨”´  2. u?ƒ  3.¾Q´w ÑL S}Öu=Á |  |
| 316 | KØÁo lØ` 315 SMf­ 1 ŸJ’& ¾ƒ—¬ ¨”´; | 1. ›«~  2. ¡„  3. cÙ  4. Ÿv  5. K?L |  |
| 317 | KØÁo lØ` 315 SMf­ 2 ŸJ’& ¬H Ÿ¾ƒ ÁÑ—K<; | 1.¨”´  2.v”v  3. U”ß  4.Ñ<ÉÕÉ |  |
