# Supplementary figures and images for: Epidemiology of Schistosoma mansoni infection and associated risk factors among school children attending primary schools nearby rivers in Jimma town, an urban setting, Southwest Ethiopia
Source: PLoS One. 2020 Feb 27;15(2):e0228007. doi: 10.1371/journal.pone.0228007 (PMC7046261; doi:10.1371/journal.pone.0228007)

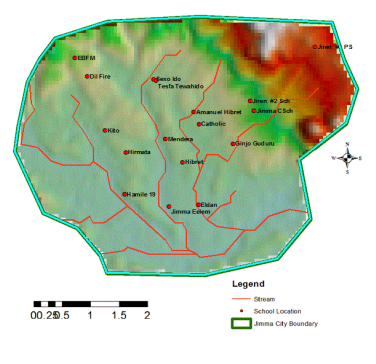


**M**ap of Jimma town with indicating schools and rivers/streams around the schools and town

Supplement: S1 File — (DOCX) [file pone.0228007.s002.docx]
