# Supplementary material for: Epidemiology of Schistosoma mansoni infection and associated risk factors among school children attending primary schools nearby rivers in Jimma town, an urban setting, Southwest Ethiopia
Source: PLoS One. 2020 Feb 27;15(2):e0228007. doi: 10.1371/journal.pone.0228007 (PMC7046261; doi:10.1371/journal.pone.0228007)
Supplement: S2 File — (DOCX) [file pone.0228007.s003.docx]

**Supporting information 2**

**Written consent to participate**

**Explanation on procedures and conditions of agreement**

We are from Jimma University, Institute of health, Faculty of Health Sciences, School of medical laboratory sciences. I am here to study about *S. mansoni* infection prevalence.

The objective of the study is to assess epidemiology of *S. mansoni* and its associated risk factors the n among school children in the selected primary schools nearby rivers in Jimma town. The information generated from this study will provide the current status of intestinal *S. mansoni* in the selected schools in Jimma town. I am asking you to participate in the study for *S. mansoni* investigation. The investigation will involve collection of feces for parasitological examination and interview through pre-structured questionnaire for demographic and associated risk factors for *S. mansoni* infection and if the result of investigation is positive for *S. mansoni* infection, you will be treated with appropriate drug. Dissemination of the results will be forwarded, we assure you the confidentially of all collected information in the questionnaire and fecal examination. Additionally it is your right to withdraw from this study if you are not interested to participate in the study. Finally, if you have understood the explanation very well I am asking you kindly to participate in this study, and put your signature below. It is with full understanding of the situation that I agreed to give the informed consent voluntarily to researcher. I agree that I am contributing to prevention and control of the disease my fellows and myself by participating in the research.

Signature (participant) __________ Date___________

Signature (Investigator) _________ Date __________

Thank you for your participation
