# Supplementary material for: Epidemiology of Schistosoma mansoni infection and associated risk factors among school children attending primary schools nearby rivers in Jimma town, an urban setting, Southwest Ethiopia
Source: PLoS One. 2020 Feb 27;15(2):e0228007. doi: 10.1371/journal.pone.0228007 (PMC7046261; doi:10.1371/journal.pone.0228007)
Supplement: S3 File — (DOCX) [file pone.0228007.s004.docx]

**Supporting information 3**

**Guca wadda( Written consent translated to local language)**

**Seensa**

Gucawaligalte matiin baraata qorraannoo kessaatti hiramchisiisuuf fedhii qabachuu isaan ittin walgalte isaan ibsan.

Kayyoon guddan qorannichaa haalli tamsa’ina fi ciminaa dhukkuba Bilaaharziiyaa mar’imaanii ijoollee mannen baruumissa nanno lagatti dhi’ataanii jiran irrattii sakatta’uufi.

Akkasumas gaafannoon kun wantoota dhukkuba bilaaharziiyaa mar’imaanii namatti fidan irratii hubanno fi ilaalchaa uumatin qabu madaaluuf kan qopha’eedha.Kana keessatti hirmannan keessan baa’ee barbachisaadha. Yoo waali galttan, fakkisa boolii guddaa qoodaa laastikii kubbaayya qoopha’ee jiru kanatti isin irra argachuun barbada,kunis jiirachuu bilaharziiyaa mar’immanii keessaa sakkata’uf ta’a. Qoranno kana keessatti hirmachuun keessan balaa isinttii fiduu hinqabuu.Yoo dhuukubin kun isin keessatti argaame qorchaa isaa tola fudhatu. Odeefannon isin kennitan dhimma qorannoo kanaatiif qofa kan fayyadamnnu fi qaama birootti kan hin himamne wan ta’eef soda tokko illee hinqabatinaa. Ittidabalees qorannicha keessati hirmaachuu dhiisunii fi yeroo barbaaaddan addaan kutuun mirga keessani.Hirmaannaa keessaniif isin galateeffanna.

Qorannicha keessatti hirmaachuuf fedhii guutuu qabdduu?

1. Eyyee 2. Lakki

Guyyaa _______________

Maqaa gaafatichaa ________________

Maqa to’ataa ___________________

Maqaa mana Barumssa ___________________

**Gallatooma hirmannaa keessaniif!!!**
