# Supplementary material for: Epidemiology of Schistosoma mansoni infection and associated risk factors among school children attending primary schools nearby rivers in Jimma town, an urban setting, Southwest Ethiopia
Source: PLoS One. 2020 Feb 27;15(2):e0228007. doi: 10.1371/journal.pone.0228007 (PMC7046261; doi:10.1371/journal.pone.0228007)
Supplement: S1 Data — (DOCX) [file pone.0228007.s005.docx]

**LABORATORY PROCEDURE OF KATO- KATZ EGG COUNTING TECHNIQUE**

1. Place a small amount of fecal material on scrap paper and press the small on top.
2. Scrap with flat-sided spatula across the upper surface of the screen to collect the sieved feces.
3. Place template with 41.7mg hole size on the center of a microscope slide and add feces from the spatula so that the hole is completely filled. Pass the side of the spatula over the template to remove the excess feces from the edge of the hole.
4. Remove the template carefully so that the cylinder of feces is left over the slide.
5. Cover the fecal material with the pre-soaked cellophane strip.
6. Invert the microscope slide and firmly press the fecal sample against the cellophane strip on another microscope slide or smooth hard surface.
7. Carefully remove the slide by gently sliding it side ways to avoid separating the Cellophane strip or lifting it off.
8. For all except hookworm eggs, keep the slide for one or more hours at ambient temperature to clear the fecal material prior to examination under the microscope.
9. Examine the smear systematically and count the number of eggs of each species of the parasite. Multiply the number of eggs a factor depending on size of the template to report egg per gram of stool.
