# Supplementary material for: Epidemiology of Schistosoma mansoni infection and associated risk factors among school children attending primary schools nearby rivers in Jimma town, an urban setting, Southwest Ethiopia
Source: PLoS One. 2020 Feb 27;15(2):e0228007. doi: 10.1371/journal.pone.0228007 (PMC7046261; doi:10.1371/journal.pone.0228007)
Supplement: Attachment — Submitted filename: Response to reviewer comments.docx [file pone.0228007.s006.docx]

Response to reviewer comments on Epidemiology of *Schistosoma mansoni* infection and associated risk factors among School Children attending Primary Schools nearby Rivers in Jimma Town, an urban setting, Southwest Ethiopia

| Academic editor and reviewers comments | Response |
| --- | --- |
| Academic editor   - Significance of probabilities P<0.000 - Survey questions or questionnaires used in the study to assess participant behaviors (eg. swimming, river contact, washing) - overlapping some text in previous publications | - The comment is accepted and corrected to P<0.0001 in the Table 3 between line 307 and 308 - The comment is accepted and survey questionnaire both in local language and English version attached as supporting information - With regard to overlapping of some text in one of the publication I am the author and the study area is different from the current even the objective is different meaning the current one is to assess the distribution of *S. mansoni* among school children near to water bodies in an urban setting where as the previous one was done in the rural areas. The risk factors where ever it is the same. With others publications mentioned I used the figures from the articles for review and they were done somewhere else. Overall the current research focus on school children near to water bodies in the town and the assess the effect of water on *S. mansoni* we have published one articles before in the town which reported prevalence of *S. mansoni* in which the risk factors weren’t assessed and second there is variation among the schools in prevalence and now we have focused on school children near to water bodies to see the effect of water bodies and association with *S. mansoni* |
| Reviewer #1   - Epidemiological study issue - Sample size issue - Results compared with previous study done in the area - With regards to no unique findings with references mentioned - With regards to single stool samples - Long paragraph from line 85-97 in the introduction part - Reference too many - English should be improved | - Yes we have done epidemiological study in the town and we have reported prevalence data and associated risk factors for *S. mansoni* infection. With regard to the survey it isn’t simple now days mass drug administration was implemented in different areas of the country and our study was done in urban settings and in this areas in most cases mass drug administration wasn’t implemented at the time of data collection and it gives hint for Jimma town health office to implement mass drug administration. - With regard to sample size we can’t say the sample size is too small as we determined sample size based sample size calculation formula. The other issue we can’t screen all school children because of resource and time and human power also. Thus why we have used some previously published article and used P value 26.3% from the research done in communities living in Jimma town near by three rivers in the town. So we have followed recommended procedure for determining sample size for doing research. - Reference 40 the research was done in the rural area of the zone on intestinal helminthiasis surrounding districts hydro electric dam in which the prevalence was not compared with the current study which was 2.1% vs 28.7%. Even the school children considered as low risk versus moderate risk. - Reference 41 the study objective is different from the current one and there was repeated infection from registration book in the health facility and we suspect drug resistance and we did drug efficacy meanwhile we determine prevalence but the study area was rural vs urban in which the setting different. - The same is true for reference 42 except we have assessed the risk factors in the same area with 41 with slight difference with prevalence. - Reference 43 the study was in the same area in the town and only prevalence data was reported and the risk factors weren’t assessed. In the previous study we have tried to include all the school children in the town and we have reported there great variation in prevalence of *S. mansoni* among the schools and those schools near to water bodies were more infected and thus why we took this five school and we have tried to report both risk factors and prevalence and the prevalence was different 8.4% in previous and 28.7% in the current. Even had it been more samples were screened the prevalence was may be more than this. - Yes we have collected single stool sample from school children but we have prepared double kato katz from single stool samples based on WHO guideline recommendation for screening of intestinal parasitosis. We have used double Kato katz to decrease the chance of missing the ova and increasing the rate of prevalence as per recommendation of WHO guideline. - The comment is accepted and I have tried to shorten the lines. - There is no restriction of reference and others in the guideline and all the references I have used are relevant with our manuscript for discussion and even for literature review. - The comment is accepted and I have tried to improved it |
| Reviewer #2 |  |
| - Tefera and his colleagues investigation on *S. mansoni* infection and its associated factors among school children in Jimma town | - The comment is accepted but I couldn’t find the article thus why I didn’t include either in review literature or discussion. |
| - CDC guideline three samples examined for three different days - About other helminthes and protozoa detected in the stool - About discussion - About schistosomes italics - Sex - Hepatomegally and splenomegally | - Examination of stool for three days on every other day is recommended for diagnostic purpose not for screening of large sample size. We have used WHO guideline published on 2014 which recommended taking single stool sample and examine using single thick Kato Katz for *Schistosoma mansoni* and soil transmitted helminthiasis and we have used double Kato Katz to increase sensitivity. It is most difficult to take three samples from each student at day interval of three days due to resources and time too. Therefore the comment raised by reviewer is used for diagnostic purpose not for research case. - The comment is accepted and the data with specific Species of parasites were included in the manuscript - In the first paragraph of discussion I have tried to discuss our findings, then I have discussed with other findings done before either in the country or abroad that is what we have to do under discussion and up to my understanding the discussion is well discussed with similar research done on school children. - The comment accepted and it is italicized and corrected - The comment accepted and corrected then changed to gender - The comment accepted and corrected as hepatomegaly and splenomegaly line 189 and 190 |
| Reviewer#3 |  |
| - About associated determinants factors | - The comment is accepted and changed to risk factors |
| - School representativeness - Clinical manifestation specific with headache - Map of Jimma town with schools | - Yes these schools were selected based on proximity to water bodies with near the schools and river crossing the town and schools were purposely selected but the students were by systematic random sampling technique which has no bias on students selection with proportional allocation was made to each school. Thus the result derived from the schools can be representative - The school environment is close to the water bodies in which the students have contact with these water bodies during their vacation and playing in the field in the compound and the snail serving as intermediate host is available in water bodies and identified as they are infected. Thus in the surrounding environment there are risk factors for infection for *S. mansoni*. In the previous study we have reported prevalence *S. mansoni* in the town but we didn’t assessed the risk factors and now we included assessing risk factors focusing on schools closed to water bodies. - The comment accepted and the headache deleted between line 188 and 189 - I have attached jimma town map with school location |
|  |  |
